# Neues vom Grand Hotel Abgrund. Der Paradigmenwechsel vom Kosmopolitismus zur Kosmo-Politik

**DOI:** 10.1007/s12286-021-00479-4

**Published:** 2021-04-16

**Authors:** Claus Leggewie

**Affiliations:** Zentrum für Medien und Interaktivität, Ludwig Börne-Professur, Justus-Liebig-Universität Gießen, Ludwigstraße 34, 35390 Gießen, Deutschland

**Keywords:** Kosmopolitismus, Anthropozän, Kosmo-Politik, Umweltpolitik, Globalisierung, Planetares Denken, Umweltrecht, Transnationalismus, Konstitutionalismus, Nicht-menschliche Natur, Cosmopolitanism, Anthropocene, Cosmo-Politics, Environmental Politics, Globalization, Planetary Thinking, Environmental Law, Transnationalism, Constitutionalism, Non-Human Nature

## Abstract

Die altehrwürdige, stets bestrittene und leicht angegraut wirkende Tradition des Weltbürgertums (Kosmopolitismus) steht (I) vor drei neuen Herausforderungen: (a) postkolonial, insofern die westlichen Ursprünge universaler Ideen auf der Hand liegen und daraus eventuell Verengungen und Einseitigkeiten folgen, (b) als Eliten-Projekt, das die breite Bevölkerung nie erreicht hat bzw. diese ignoriert, gepaart (c) mit Einwänden von kommunitaristischer Seite, wonach alle Vorstellungen von Zugehörigkeit, Solidarität und Hospitalität in lokalen Gemeinschaften geerdet sein müssen. Die Idee des Kosmopolitismus kann sich diesen Herausforderungen stellen, wenn sie (II) stärker implementiert und operationalisiert wird: Dazu tragen Ansätze eines „globalen Konstitutionalismus“ bei, der über den Nationalstaat als überkommener Stütze von Regierung und kollektiver Identität hinausreicht und Problemlagen angeht, die diesen Souveränitäts- und Identitätsrahmen gesprengt haben. Im Zeitalter des Anthropozän (III) ist eine Erweiterung des Kosmopolitismus angebracht, nämlich die überfällige Einbeziehung der belebten und unbelebten Natur als einem virtuellen Mit-Akteur internationaler Beziehungen. Diese konzeptionelle und operative Revision kosmopolitischer Ideen mündet in eine übergreifend planetare „Kosmo-Politik“.

## Abstract (en)

The time-honored, always contested and slightly graying tradition of cosmopolitanism faces (I) three new challenges: (a) postcolonial, insofar as the Western origins of universal ideas are obvious and narrowing and one-sidedness may follow from this, (b) as an elite project that has never reached or ignores the broad population, coupled (c) with objections from the communitarian side that all notions of belonging, solidarity, and hospitality must be grounded in local communities. The idea of cosmopolitanism can face these challenges if it is (II) more implemented and operationalized: Approaches to “global constitutionalism” that reach beyond the nation-state as an outdated support for government and collective identity and address problem situations that have broken this framework of sovereignty and identity contribute to this. In the age of the Anthropocene (III), an extension of cosmopolitanism is appropriate, namely the overdue inclusion of animate and inanimate nature as a virtual co-actor of international relations. This conceptual and operational revision of cosmopolitan ideas results in overarching planetary “cosmo-politics.”

## Besprochene Bücher

### Kritischer Kosmopolitismus

Brudermüller, Gerd, Daniela Demko und Kurt Seelmann (Hrsg.) 2019. *Kosmopolitismus in einer globalisierten Welt*, Würzburg: Königshausen & Neumann, 201 Seiten, Paperback 25,00 €

Hahn, Henning. 2017. *Politischer Kosmopolitismus. Praktikabilität, Verantwortung, Menschenrechte*, Berlin: De Gruyter, 256 Seiten, Hardback 99,95 €, e‑book 102,95 €

Nida-Rümelin, Julian, Detlef von Daniels und Nicole Wloka (Hrsg.) 2019. *Internationale Gerechtigkeit und institutionelle Verantwortung*, Berlin: De Gruyter, 426 Seiten, Hardback 82,95 € (Interdisziplinäre Arbeitsgruppen, Forschungsberichte Bd. 41)

DeArmey, Michael H. 2020. *Cosmopolitanism and the Evils of the World*, London: Palgrave/Macmillan, 310 Seiten, Hardback 88,39 €, e‑book 71,86 €

Delanty, Gerard (Hrsg.) 2019. *Routledge International Handbook of Cosmopolitanism Studies*, 2. Auflage London: Routledge, 664 Seiten, Hardback 205,56 €, Paperback 51,99 €, e‑book 28,84 €

Cicchelli, Vincenzo und Sylvie Mesure (Hrsg.) 2020. *Cosmopolitanism in Hard Times*, Leiden: Brill, 420 Seiten, Hardback 240,00 €.– (International Studies in Sociology and Social Anthropology, Bd. 13)

### Auf dem Weg zu einer globalen Umweltverfassung?

Atilgan, Aydan 2018. *Global Constitutionalism. A Socio-Legal Perspective*, Cham: Springer, 315 Seiten, Paperback 119,67 € (Beiträge zum ausländischen öffentlichen Recht und Völkerrecht, Bd. 275)

Lang, Anthony F. jr., Wiener, Antje (Hrsg.) 2017. *Handbook on Global Constitutionalism*, Oxford: Edward Elgar, 457 Seiten, Hardback 225,56 € (Research Handbooks on Globalisation and the Law)

Suami, Takao, Peters, Anne, Vanoverbeke, Dimitri, Kumm, Mattias (Hrsg.). 2018, *Constitutionalism from European and East Asian Perspective*, Cambridge/UK: Cambridge University Press, 607 Seiten Hardback 131,13 €, Paperback 42,24 €, e‑book 22,47 €

Kotzé, Louis J. 2016. *Global Environmental Constitutionalism in the Anthropocene*, Oxford: Hart Publ., 281 Seiten, Hardback 77,99 €, Paperback 36,10 €, e‑book 22,91 €

Heyvaert, Veerle, Duvic-Paoli, Leslie-Anne (Hrsg.) 2020. *Research Handbook on Transnational Environmental Law*, Chattenham: Edward Elgar, 416 Seiten, 210,07 € (Research Handbooks on Globalisation and the Law)

### Posthumane Internationale Beziehungen?

Eroukhmanoff, Clara et al. (Hrsg.) 2017. *Reflections on the Posthuman in International Relations: The Anthropocene, Security and Ecology*, E‑International Relations, o. O. 131 Seiten, 18,56 €

Castro Pereira, Joana/Saramago, André (Hrsg.) 2020. *Non-Human Nature in World Politics. Theory and Practice*, Cham: Springer. 352 Seiten, Hardback 103,99 €, e‑book 85,59 €

Biermann, Frank/Lövbrand, Eva (Hrsg.) 2019. *Anthropocene Encounters: New Directions in Green Political Thinking*, Cambridge UK: Cambridge University Press, 262 Seiten, Hardback 39,30 €, Paperback 43,70 €, e‑book 30,71 €

## Kritischer Kosmopolitismus

ME VELLE CIVEM ESSE TOTIUS MUNDI NON UNIUS OPPIDI^1^, prangt auf der Rückseite einer niederländischen 25 ECU-Münze. Schöne Idee, schlechte Utopie? Die altehrwürdige, stets bestrittene und vielen angegraut, manchen auch totalitär anmutende Idee des Weltbürgertums, hier im Wunsch des Humanisten Erasmus von Rotterdam, steht vor drei Herausforderungen:

- *erstens* aus postkolonialer Perspektive wegen der Einseitigkeiten und Blindstellen, die eventuell aus den westlichen Ursprüngen dieser universalen Idee folgen;
- *zweitens* als Eliten-Projekt, das die Massen nie erreicht hat und manifeste Ungleichheiten und strukturelle Ungerechtigkeiten in der Weltgesellschaft ignoriert;
- *drittens* durch Einwände von kommunitaristischer Seite, die Leitkonzepte wie Zugehörigkeit, Solidarität und Hospitalität vornehmlich in lokalen Gemeinschaften verankert sieht.

Damit ist der Kosmopolitismus keineswegs passé, wie seine kritische Affirmation durch so bedeutende GegenwartsphilosophInnen wie Seyla Benhabib (2016), Kwame Anthony Appiah (2009), Martha Nussbaum (2010) und andere belegt; getestet wird dieses „noble, but flawed ideal“ (Nussbaum 2020) an aktuellen Notlagen wie globalen Flüchtlingsbewegungen (Marsili und Milanese 2019). Die immer wieder ergiebige Inspektion der Ahnenreihe kosmopolitischer Ideen- und Ratgeber von Sokrates und Diogenes über Cicero und Marc Aurel bis Grotius und Kant erfolgt heute „ohne Illusionen“ (Benhabib 2016) über die Zahnlosigkeit eines wohlmeinenden Gesinnungs-Weltbürgertums und die Grenzen des fragmenthaften Institutionen-Weltbürgertums. Der aus Ghana stammende, in Princeton lehrende Philosoph Appiah mahnt, Entscheidungen, die auf den ersten Blick kosmopolitisch geraten scheinen, genauer zu durchdenken (2009, S. 199 ff.) Die in Yale tätige Seyla Benhabib setzt auf Jurisgenerativität, also die „Fähigkeit des Rechts, ein normatives Bedeutungsuniversum zu schaffen, das sich von der ‚Herkunft formaler Gesetzgebung‘ oft freimachen kann, wodurch Sinn und Reichweite des Rechts selbst ausgeweitet werden“ (2016, S. 42). Menschenrechtserklärungen und -verträge erlauben „neuen Akteuren“ (das sind Frauen, Minderheiten und Staatenlose), in die sich weitende (Welt‑)Öffentlichkeit vorzudringen und überzeugende Vokabulare für ihre gerechten Forderungen zu finden. In diesem diskursiven und deliberativen Kontext könnten sich Benhabib zufolge „Prozesse kaskadenförmiger demokratischer Iterationen einen Weg bahnen“ (2016, ebd.) Die in Chicago lehrende Philosophin Martha Nussbaum verfeinert den „Fähigkeitenansatz“ (2020, S. 15 f.) des Wirtschaftswissenschaftlers Amartya Sen und beharrt, stärker als in früheren Arbeiten, auf der zentralen Rolle der Nation als größte Einheit menschlicher Autonomie und auf den Nationalstaaten als bestmöglichen Organisatoren einer annähernd gerechten Verteilung endlicher Ressourcen und „überflüssigen Reichtums“ zwischen armen und reichen Gesellschaften, ein ursprünglich von Henry Thoreau in „Walden“ verwendete Formel. Dabei rückt sie auch die nicht-menschlichen Lebewesen ins Licht, die in der antiken Tradition wie im politischen Liberalismus vernachlässigt wurden (s. Abschnitt *Posthumane Internationale Beziehungen?*).

Die Lücken kosmopolitischen Denkens angesichts aktueller Herausforderungen (Migration, Klimawandel, Terror) indizieren nun erneut eine ganze Reihe von Studien zum „politischen Kosmopolitismus“ in einer, wie es dann stets tautologisch heißt, „globalisierten Welt“. Wie kann die Welt den „Anderen“ besser gerecht werden: Armen, künftigen Generationen, nicht-menschlicher Umwelt? Die Kasseler Habilitationsschrift des Philosophen Henning Hahn misst eine Theorie globaler Gerechtigkeit an den Kriterien Akzeptabilität und Praktikabilität. Damit sind die hohen Standards (und der schlechte Utopismus) des moralischen und juridischen Kosmopolitismus an Maßstäben einer Politikpraxis zu messen, die offenbar unüberwindlich von Machterwerb und Machterhaltung charakterisiert ist; doch wie kann eine solche „Realpolitik“ aussehen, die sich nicht dem weit schlechteren Realismus des Status quo anbiedert und vor der Willkür von Großmächten und Autokraten einfach kapituliert? Für eine realistische, plurale und konsensfähige Konzeption der Menschenrechtspolitik findet Hahn „empirische Spuren“ (2017, S. 215 ff.) in bereits bestehenden supra- und transnationalen Regimen, bei aufgeklärten einflussreichen Wirtschaftsakteuren und in zahlreichen Netzwerken von Einzelpersonen, die ihr Alltagsverhalten menschenrechtlich habitualisieren. Analog zu dem Konstrukt „transitorischer Gerechtigkeit“ (vgl. etwa Archibugi und Pease 2018) skizziert Hahn einen Kosmopolitismus, der eine „schöne“ Theorie in „schäbige“ politische Praxis umzusetzen versteht.

In eine ähnliche Richtung zielen Beiträge zum Symposium des Instituts für angewandte Ethik in Bad Dürkheim 2018. Der Philosoph Andreas Niederberger (Universität Duisburg/Essen) nimmt im Einleitungsbeitrag „Was bedeutet Kosmopolitismus im 21. Jahrhundert? Bilanz und Perspektiven“ (S. 9–56) eine Umkehr der Beweislast vor: Ein kosmopolitisches Programm müsse verstehen, wie es sich „in einer eventuell ihm zuwiderlaufenden Welt zur Geltung bringen kann“ und daran messen lassen, „dass es möglicherweise keine Selbstverständlichkeit eines Vorrangs des Völkerrechts vor nationalem Recht, eines Gewaltverbots in den internationalen Beziehungen oder der Geltung wenigstens einiger grundlegender Menschenrechte gibt. Und auch die Vorzugswürdigkeit multilateraler Koordinationen und Kooperationen gilt nicht (mehr) unbefragt“ (S. 54 f.) Ein abgeklärter Kosmopolitismus des 21. Jahrhundert geht dann rundum kontestatorisch vor, indem er Denk‑, Argumentations‑, Rechtfertigungs- und Handlungsweisen der Kritik unterzieht, bei denen einerseits „der normative Pluralismus und die Konflikthaftigkeit unseres Universums, auch in normativer Hinsicht nicht anerkannt wird“, zum anderen Personen und Gruppen, die meinen „auf der Basis je eigener normativer Einsichten über die normative Autorität anderer entscheiden und agieren zu dürfen“. Entscheidend ist dann, ob und „wie diejenigen, die global Strukturen, Maßnahmen oder Handlungen anderer primär unterworfen werden, sich in der Entscheidung über sie zu Gehör bringen konnten und können“ (S. 52 f.).

Die stets suboptimale Bearbeitung des „Migrationsproblems“ liefert diesbezüglich eine Art von Lackmustest. Die emblematische Figur des Flüchtlings durchzieht auch einige Beiträge einer Tagung der Berlin-Brandenburgischen Akademie der Wissenschaften zum Komplex „Internationale Gerechtigkeit und institutionelle Verantwortung“. Der Herausgeber Julian Nida-Rümelin (München) betrachtet sich als Kosmopolit und argumentiert – kontestatorisch also! – gegen offene Grenzen. Das begründet er nicht kommunitaristisch-nationalistisch (also etwa damit, dass eine ethnische Gemeinschaft zu viel Fremdheit nicht aushalten könne), sondern republikanisch-kosmopolitisch, also im Einklang mit einer föderalen Weltordnung mit institutionellen Strukturen (darunter: Grenzen), die effektives politisches Handeln und Entscheiden auf allen Ebenen ermöglichen soll. Grenzen bilden für Nida-Rümelin den Schutzraum für individuelle und kollektive Autonomieansprüche und widersprechen dem Gleichheits- und Gleichbehandlungsgrundsatz nicht, wenn ihre Existenz und ihre Aufrechterhaltung in Fällen krasser sozialer Ungleichheiten, von Kriegs- und Bürgerkriegsfolgen und Naturkatastrophen ethisch begründet werden kann. Eine globale Zivilgesellschaft, betont er, entstehe nicht durch Errichtung eines gesonderten Weltstaats, sondern gewissermaßen von unten „in Fortsetzung bestehender Bindungen und Interaktionen, die im globalen Maßstab ephemerer werden und eine Normativität voraussetzen, die partikulare Bindungen überwölbt“ (253). Dem widerspricht die Migrationspolitik reicher Länder, die nur humanitär redet und protektionistisch handelt.

Der Kosmopolitismus spielt für die Disziplin der internationalen Politik eine naturgemäß wichtige Rolle (vgl. Beck und Grande 2004; Zürn 2011). Die Berliner Fachvertreter Wolfgang Merkel und Michael Zürn identifizieren in dem vorliegenden Band (S. 67–101) eine politische Spaltung zwischen wirtschaftlichen wie kulturellen Gewinnern und Verlierern der Globalisierung, die zu einer Repräsentationslücke in den europäischen Parteiensystemen geführt habe. „Es hat sich in den letzten zwei bis drei Jahrzehnten eine anwachsende Gruppe von Bürgern gebildet, die sich weder ökonomisch noch diskursiv oder kulturell von den etablierten Parteien repräsentiert fühlt. In diese Repräsentationslücke haben sich in West- wie Osteuropa die Rechtspopulisten eingenistet. Die Dynamik ihrer zunehmenden Wahlerfolge und die Tatsache, dass in der einstigen demokratischen Vormacht der Welt mit Trump ein Präsident gewählt wurde, der sich rechtspopulistischer Techniken bedient und nationalistische Inhalte vertritt, wird die neue Konfliktlinie weiter verstetigen“ (Merkel und Zürn 2019, S. 97). Zwar treffe die kommunitaristische Kritik das empirisch-institutionelle Manko kosmopolitischer Ideen, also die Festlegung eines Schwellenwertes von „Betroffenheit“ und daraus folgend des Ausmaßes von Solidarität und Umverteilung sowie nicht zuletzt die Praktikabilität supranationaler Politikentscheidungen bei Eingriffen in die nationalstaatliche Souveränität und der Öffnung von Grenzen für Personen, Waren und Dienstleistungen. Aber sie drifte aktuell vor allem in nationalistische und kulturell-identitäre Versionen von „Gemeinschaft“ ab. Um einer utopischen Überdehnung ebenso wie einer Kapitulation vor den Verhältnissen zu entgehen, rät Zürn im Verhältnis zum intergouvernementalen Modell globaler Ordnung und dem kosmopolitischen Modell der Demokratie zu „milderen“ Formen von Kosmopolitismus. „Die empirischen Bedingungen, die vom kosmopolitischen Pluralismus und kosmopolitischen Föderalismus vorausgesetzt werden, sind zwar bei Weitem noch nicht hinreichend erfüllt. Es lässt sich aber tatsächlich auf entgegenkommende Tendenzen verweisen. Dies gilt in besonderer Weise für den kosmopolitischen Pluralismus. Im Falle des kosmopolitischen Föderalismus stellt v. a. die Frage nach der Anerkennung einer zwangsbefugten internationalen Einrichtung zur Verwirklichung der Grundrechte einen Problembereich dar“ (Zürn 2011, S. 110).

Einen umfassenden Einblick besonders in die angloamerikanische Forschungsliteratur bietet das „Routledge International Handbook of Cosmopolitanism Studies“, herausgegeben von dem an der University of Sussex lehrenden Soziologen Gerard Delanty. Es resümiert Ergebnisse sämtlicher mit Cosmopolitanism befasste Disziplinen, thematisiert die produktive Spannung zwischen normativen (philosophischen) und empirischen (anthropologischen und soziologischen) Ansätzen und greift weit über die „alteuropäischen“ (antiken wie modern-liberalen) Fassungen des Kosmopolitismus in andere Kontinente aus. Realgeschichtlich hat die sukzessive Globalisierung dem kosmopolitischen Denken eine wirklich globale Basis verliehen – und das scheint jetzt zu einem gewissen Abschluss gelangt zu sein. Gegen die nationalistische Herausforderung formuliert Delanty einen (selbst-)kritischen Kosmopolitismus, der lokalen Gemeinschaften die Chance der Relativierung und Reflexivität des eigenen (bornierten) Standpunkts bietet, ohne dabei in jenen uferlosen Relativismus zu verfallen, der dem aktuell zu beobachtenden Streben nach Gruppen-Identität und der exzessiven Betonung ethnisch-religiöser Diversität eigen ist.

Dabei stellt gerade ein derart „verwurzelter“ Kosmopolitismus eine wichtige Referenz für Minderheiten dar und kann ein Motor von Demokratiebewegungen sein. In Abwandlung einer berühmten Formel könnte man sagen, dass der Kosmopolitismus von Voraussetzungen lebt, die er selbst nicht hervorgebracht hat, dass aber diese kommunitären Wurzeln ohne übergreifende Rahmung leicht antagonistisch und polarisierend verkümmern. Auch der von den SoziologInnen Vincenzo Cicchelli und Sylvie Mesure (Paris) herausgegebene Band „Cosmopolitanism in Hard Times“ rekapituliert die Genese und Etablierung kosmopolitischer Ideen und buchstabiert aktuelle Erfahrungen und Herausforderungen durch, hier auch mit einem Blick auf den Einfluss des Kapitalismus. Die 27 bzw. 45 Beiträge können hier nicht im Einzelnen gewürdigt werden, bieten aber in der Summe einen durchweg kompetenten und beinahe vollständigen (s. unten) Überblick über eine, so der Eindruck des Rezensenten, bei aller Bringschuld keineswegs veraltete Idee, die der Wiederbelebung und dabei auch einer thematischen Erweiterung bedurfte.

In diese Richtung hat der an der University of Southern Mississippi lehrende Moralphilosoph Michael H. DeArmey das Kantsche Konzept vom Bösen fortgeschrieben. Heutige „Evils“ sind für ihn die Zerstörung der Biosphäre, fortbestehende Völkermorde, neue Formen der Sklaverei (u. a. in der Gestalt von Kindersoldaten), die Anwendung von Folter und der Terrorismus. Die stets unzureichende Bekämpfung dieser Übel durch Nationalstaaten und die Ohnmacht internationaler Organisationen erfordere eine starke kosmopolitische Antwort, die DeArmey in einer „Liste kosmopolitischer Empfehlungen“ (S. 285 ff.) resümiert. Sie beginnt mit einer radikalen Reform der Vereinten Nationen wie der Hochstufung der UN-Charta zur Weltverfassung, der Einrichtung eines Umweltrates mit den analogen Kompetenzen des Sicherheitsrates und einer Volksversammlung als zweiter Kammer. Es folgen Vorschläge zur Aufnahme von Flüchtlingen, zur Ächtung sklavenartiger Abhängigkeiten, zur Waffenkontrolle, zum Bann der Folter und zur Bekämpfung von Hasskriminalität sowie eher kontroverse Anregungen zur Geburtenkontrolle und „holistischen“ Rekonstruktion gescheiterter Staaten. Interessant ist die Lenkung kosmopolitischer Aufmerksamkeit auf globale Unternehmen, deren CEOs – genau wie hohe Regierungspersonen – künftig eine Führungslizenz vorweisen sollen. Die Befugnisse des neu zu schaffenden UN-Umweltrates COPRE (Council for the Preservation and Restoration of the Environment) werden über bloßes Monitoring ausgedehnt auf effektive Verbote von nicht recycelbarem Plastik, der Abholzung von Regenwäldern, der Verwendung von Pestiziden und der Herstellung von Automobilen mit Verbrennungsmotoren. Empfohlen wird ferner ein Moratorium zur Vermeidung der Überfischung, die Abschaltung von Kohlekraftwerken, die Umstellung der Haushalte auf Wind- und Solarstrom, die Rekuperation verlassener Innenstädte zur Vermeidung von Landschaftszersiedelung und Bodenversiegelung. Zur Verlebendigung kosmopolitischer Prinzipien in der Weltgemeinschaft schlägt der Autor interkulturelle Clubs, Flüchtlingsstädte, Friedenskorps und Austausch- und Sprachlehrprogramme und eine Kunstolympiade, Habitate mit bedrohten Arten sollen durch lokale Naturschutzvereine adoptiert werden. DeArmey schließt in sein an Immanuel Kant angelehntes Konzept der Würde und Gastfreundschaft ausdrücklich nicht-menschliche Lebewesen ein. Kants Konzept des (radikal) Bösen wird von ihm aber kaum reflektiert, da zum Beispiel Hannah Arendts Lektüre im Licht des Holocaust bei deArmey fehlt.

## Auf dem Weg zu einer globalen Umweltverfassung?

*The proof of the pudding is the eating.* In vor allem zwei Richtungen ist die „schöne Idee“ des Kosmopolitismus im 20. Jahrhundert institutionell verankert und implementiert worden – in Richtung auf eine Transnationalisierung liberaler und sozialstaatlicher Verfassungsgrundsätze und in Gestalt von Institutionen „globalen Regierens“, das nationale Grenzen überschreitet und dabei nicht imperial-autoritär vorgeht, sondern demokratisch legitimiert ist. Seit der Neuzeit sind Rechtsgarantien stets an den Nationalstaat gebunden, Politik, Öffentlichkeit und Wissenschaft akzeptieren diesen Konnex als eine Standardannahme, auf welche die Durchsetzung von Menschenrechten letztlich angewiesen bleibt. Jedes von der Sache her über staatliche Grenzen hinausdrängende Policy-Feld, so auch die „Umwelt“, scheint in diese Souveränitätsschranke verwiesen; auch wenn der grenzüberschreitende Charakter von Problemlagen und Normen des „Umweltschutzes“ anerkannt wird, agieren die Akteure weiter im Horizont ihrer jeweiligen nationalen Verfassungsordnungen. Nur die Europäische Union sorgte mit ihrer speziellen, weder als Staatenbund noch als Bundesstaat einzuordnenden Mehrebenen-Architektur für eine Aufweichung eherner Souveränitätsvorbehalte.

Gemessen daran, ist die Entwicklung eines „Global Environmental Constitutionalism“ hoch ambitioniert. Kann man eine globale wirksame Umweltverfassung schaffen? Verfassungen sind formuliert und verankert worden, um politische Macht zu zähmen und Alleinherrscher und Oligarchen an Regeln zu binden (Grimm 2016). Dazu werden Legalität und Legitimität staatlicher Ordnungen durch Parlamente und Gerichte kontrolliert, die Gewalten geteilt und persönliche Grundrechte der zu Staatsbürgern und -bürgerinnen aufgestiegenen Untertanen garantiert. Dieser als Konstitutionalisierung bezeichnete Prozess, der eine Fülle (kulturell geprägter s. unten) Varianten hervorgebracht hat, war dynamisch und unterstützte die Demokratisierung, beginnend mit der Institutionalisierung freier, allgemeiner und fairer Wahlen, dem Aufbau rechtsstaatlicher Institutionen und die Übernahme politischer Kontrolle durch Verfassungsgerichte, wobei Verfassungsgebung und -inhalte stets mit Mehrheitsentscheidungen des Souveräns in Konflikt geraten können.

Als globalen Konstitutionalismus kann man im Anschluss daran neben der Diffusion von Verfassungsregeln in autokratisch regierte Gesellschaften die Übertragung konstitutioneller Vollmachten an transnationale Institutionen wie Internationale Gerichtshöfe und Organisationen identifizieren, die ein überstaatliches Recht kodifizieren. Eine systematische Aufarbeitung des Prozesses der globalen Konstitutionalisierung hat der jetzt an der Near East University in Nicosia tätige Jurist Aydin Atilgan vorgelegt, bei der er weniger eine vertikale Aufschichtung von Kompetenzkompetenzen als einen horizontalen und diskursiven „Transkonstitutionalismus“ am Werk sieht, also eine politisch-kulturelle Diffusion rechtstaatlicher Normen durch bedeutsame Akteure auf internationaler Ebene. Zu diesem Komplex liegt mit dem von Anthony F. Lang (St. Andrews) und Antje Wiener (Hamburg) herausgegebenen „Handbook on Global Constitutionalism“ mit 33 Beiträgen eine umfassende Problematisierung überwiegend anglophoner, aber auch in diesem Feld tätiger deutscher AutorInnen vor. Sie reicht von der Rekapitulation historischer Vorläufer über die grundlegenden Theorien der Politik- und Rechtswissenschaft bis zur Erörterung von Prinzipien, Praktiken, Institutionen (UN, ICC, EU, WTO) und Rahmenwerke und eröffnet abschließend neue Horizonte im Blick auf die politische Ökonomie und den religiösen Pluralismus einer post-westfälischen Staatenwelt.

Es ist ein Gemeinplatz geworden, dass man die Dreieinigkeit von Rule of Law, Gewaltenteilung und Demokratie nicht als Universalie voraussetzen darf, da sie klar erkennbare europäische und nordamerikanische Wurzeln haben. Ebenso essentialistisch wäre es allerdings, dem nun einfach „asiatische“ (analog „afrikanische“ etc.) Werte entgegenzusetzen oder anzufügen, wo auch „Asien“ alles andere als eine homogene Einheit von Kulturen und Normen repräsentiert. Das gilt auch für „Ostasien“, Sammelbegriff für eine bisher wesentlich durch ökonomische Kooperation verklammerte ASEAN-Region in Nord- und Südostasien, deren Verfassungsverständnis in einem von dem an der Waseda Universität Tokyo lehrenden Juristen Tako Suami und europäischen KollegInnen herausgegebenen Tagungsband „Constitutionalism from European and East Asian Perspective“ vergleichend herangezogen wird. Hält das „dicke“ Verständnis von Verfassung der Perspektivenerweiterung stand, wo muss es andernfalls ergänzt, modifiziert und revidiert werden? Kann man lokale und autochthone Elemente ohne weiteres anbauen? Kann die Integration nationaler Rechtskulturen in Europa als Vorbild für andere Regionen (wie ASEAN) dienen, deren ursprüngliche Mission ebenfalls wirtschaftlich bestimmt war? Die meisten ostasiatischen AutorInnen insistieren in dem Band mit insgesamt 18 Beiträgen auf staatlicher Souveränität und Gleichheit, ungeachtet der Wirkungen ökonomischer Entgrenzung und hegemonialer Tendenzen besonders der VR China. Von der Annahme einer simplen Transplantation ist man übergegangen zum Muster einer „Translation“, einer auf lokale Eigenheiten reagierenden Übersetzung europäischer Verfassungsgrundsätze. Die Herausgeberin, eine am Max Planck-Institut für Vergleichendes und Internationales Recht forschende Juristin Anne Peters bringt neben den zivilisationshistorischen Differenzen als kulturübergreifende Dimension die Sozialpolitik ein, die besonders für die Entwicklungszusammenarbeit und Global Economic Governance bedeutsam(er) ist, was ebenso, wie der Südafrikaner Louis J. Kotzé (422 ff.) betont, für die Umweltpolitik gilt. Die wichtigste Ost-West-Differenz, signalisieren Bin Li (2018, Peking) und Matthieu Burnay (London) in ihren Beiträgen, ist demokratiepolitischer Natur; wenn die VR China ihre Souveränität auf keinen Fall an internationale Gerichtsbarkeit abgeben möchte, stützt sich dies auf kulturelle Differenzen, es geschieht aber eher, um ein autoritäres System gegen eine autochthone Demokratiebewegung und externe Kritik an Menschenrechtsverstößen von außen zu schützen, die sich auf universalistische Prinzipien berufen. Mattias Kumm (New York/Berlin) wendet sich zu Recht gegen die postkoloniale (oder kommunitaristische) Essentialisierung kulturell-zivilisatorischer Differenzen, die Herrschaftsverhältnisse in den Kulturen übertüncht und Widerstandskoalitionen gegen Rechtsstaatsverstöße und Herrschaftswillkür erschwert. Ob eine Internationalisierung des chinesischen Verfassungsrechts mit der weiteren Öffnung der chinesischen Exportwirtschaft einhergehen wird, bleibt abzuwarten; ebenso möglich ist ein Export autokratischer Herrschaftsmethoden über die chinesische Außenwirtschaftspolitik.

Bezogen auf (je nach Betrachtungsweise engere oder weitere) ökologische Erfordernisse, die in supranationalen Konventionen zur Nachhaltigkeit wie den 17 SDG’s der Vereinten Nationen niedergelegt worden sind, kann man nun einen globalen Umwelt-Konstitutionalismus skizzieren, der nationale Verfassungsbestimmungen zum Umweltschutz aufgreift und diese transnational erweitert. Auch hier können Spannungen zwischen normativen Prinzipien, multilateralen Regulierungen und demokratischen Mehrheitsentscheidungen und Bürgerbeteiligung entstehen. Der Rechtsstaat, dem originär die Aufgabe des Schutzes der Individuen vor staatlicher Macht und Übergriffen Dritter zukam, übernahm im 20. Jahrhundert zunehmend Steuerungs- und Gestaltungsfunktionen, darunter die Aufgabe, Schaden präventiv abzuwenden. Massiver Schaden droht nun nach allgemeiner Erkenntnis von gefährlichem Klimawandel, Artensterben und deren Folgen. Ebenso evident ist, dass deren Folgen und Begleiterscheinungen nicht „in einem Land“ einzudämmen sein werden, vielmehr multilaterale Vorkehrungen der Vorsorge und Anpassung unabdingbar sind. Diese doppelseitige Globalisierung verlangt großflächige Maßnahmen, die logistisch und finanziell nur in internationaler Zusammenarbeit möglich sind und, wo globale Gemeingüter betroffen sind, eine Intervention in nationale Angelegenheiten unumgänglich machen. Nicht länger zu rechtfertigen sind beispielsweise die von den meisten Industrienationen getätigten Subventionen in fossile Energieträger wie Stein- und Braunkohle, deren Emissionen hauptursächlich für Klimaschäden auf der südlichen Welthälfte waren und sind, so dass der „Ausstieg“ überfällig war. Dessen Regelung hat auch die Europäische Union übernommen, an deren Beginn bekanntlich die „Montanunion“ mit einem Fokus auf Kohleförderung und Stahlproduktion stand; nach langem Zögern und Hinhalten haben sich die Mitgliedstaaten auf eine sukzessive Einstellung der staatlichen Unterstützung für Kohleenergie geeinigt und Beihilfen für Kohlekraftwerke nur noch bis 2025 zugelassen, wobei Kohle-Unternehmen großzügig kompensiert und Ausnahmeregelungen etwa für Polen eingeräumt worden sind.

So wie in diesem Fall eine fossile Produktionslinie (nicht nur) im Norden globale Schäden verursacht hat, verschlechtert analog die Schließung einer für das Weltklima bedeutsamen CO2-Senke im Süden die Klimabedingungen auf der ganzen Welt, nämlich die von den Regierungen nicht verhinderte und stellenweise auch aktiv geförderte Abholzung des Regenwaldes in Brasilien, Indonesien und im Kongo. Wenn der brasilianische Präsident Jair Bolsonaro diese kaum noch anders als kriminell zu bezeichnende Politik nicht nur toleriert, sondern aktiv propagiert und durchsetzt, wäre die Überlegung naheliegend, wie dem strafrechtlich beizukommen wäre – nicht nur innerhalb Brasiliens (etwa mit dem Einklagen garantierter Rechte von Indigenen beim Internationalen Strafgerichtshof in Den Haag auf Initiative des französischen Rechtsanwalts William Bourdon^2^), sondern zusätzlich durch legale Sanktionen internationaler Organisationen und Gerichte, bei denen Nichtregierungsorganisationen Sammelklagen anstrengen könnten.

Dafür, einer nationalen Regierung in den Arm zu fallen, um überlebensnotwendige globale Gemeingüter zu schützen, bestand bisher kaum eine Handhabe. Die Frage ist, ob dem rechtssystematische oder andere Gründe entgegenstehen. Umweltgefährdungen und -schäden wie den Klimawandel ordnet auch die Rechtswissenschaft als „globale“ Umweltprobleme ein, „weil sie einerseits sowohl grenzüberschreitende, vielfach weltweite Gefahren für die Ökosysteme und die Menschen darstellen wie auch durch grenzüberschreitende Verursacherketten hervorgerufen werden und weil sie andererseits nicht durch nationalstaatliche Alleingänge erfolgreich bekämpft werden können, sondern dafür internationale Kooperationen unerlässlich sind“ (Koch und Mielke 2009, S. 403). Man kann darin, wie schon angesprochen eine „doppelte“ Globalisierung auf der Seite von Verursachern und Management erblicken, der bislang eine unzureichende Globalisierung des Umweltrechts korrespondiert. Handlungsdruck erzeugen Vorstöße der Nichtregierungsorganisationen, die sich (zum Teil durchaus regierungsnah) in die Ausformulierung von Umweltvölkerrecht eingeschaltet haben, auch die Aufnahme von Umweltaspekten in die Streitschlichtungspraxis der weit mächtigeren Welthandelsorganisation WTO.

Unverdrossen treiben renommierte Rechtswissenschaftler das globale Umweltverfassungsrecht weiter voran, darunter der schon erwähnte südafrikanische Jurist Louis F. Kotzé, der die (im Fach bisher wenig rezipierte) Anthropozän-Debatte berücksichtigt. Auch wenn er derzeit noch keine globale „constituent power“ erkennen kann, sieht Kotzé in seiner wegweisenden Monographie „ausreichende Beweise für die Existenz und allmähliche Entstehung von verfassungsartigen Merkmalen und Elementen im globalen Regulierungsraum“ (S. 131). Internationalistische (UN- und völkerrechtsbasiert), regionalistische (EU-basiert), regulatorische (vor allem seitens der WTO), zivilgesellschaftliche (NRO-zentriert) und transnationale (zwischenstaatliche) Ansätze ergänzen sich und können aus nationalen Gesetzgebungen und Rechtskulturen konvergieren lassen (S. 102 ff.) Kotzé erhofft sich einen „constitutional moment“, wenn dem Anthropozän das Potenzial einer „exceptional rule“ (S. 42) zugesprochen wird. Idealtypisch hat sich hier eine Umweltverfassung vom internationalen über das transnationale zum planetaren Recht aufgebaut, vom menschenzentrierten über das naturzentrierte Umweltrecht weiter zu einem am Ende planetozentrischen Recht (Hanusch et al. 2021).

Ein wichtiges Kompendium stellt das von den Juristinnen Veerle Heyvaert (London School of Economics) und Leslie-Anne Duvic-Paoli (King’s College London) herausgegebene „Research Handbook on Transnational Environmental Law“ dar, wobei auch hier die Fokussierung auf Methodologie und Empirie angelsächsischen Rechts zu beachten ist, die das internationale Rechtswesen ohnehin stark bestimmen. In 22 Beiträgen entsteht ein Gesamtbild über die Ausdifferenzierung eines grenzüberschreitenden Umweltrechts, das an diversen Fällen auch die für den Fall Brasilien erwähnten Klagemöglichkeiten thematisiert. Ein transnationales Umweltrecht erweitert die Möglichkeiten von Compliance und Enforcement, es führt zu einem Legitimationszuwachs nationalen Rechts, verlinkt nicht zu trennende Materien wie Klimawandel und Artensterben, ermächtigt nicht-staatliche Akteure (auch als Kläger wie im Fall Chevron/Ekuador) und stellt den notwendigen Konnex zu den Menschenrechten (ECHR) und zum Handelsrecht (WTO) her. Das alles erfordert von der Jurisprudenz eine Akzentuierung von Rechtsvergleichen und die Einbeziehung empirischer Erkenntnisse aus „abgelegenen“ naturwissenschaftlichen Disziplinen. Und es bestätigt, wie angeschlagen die aus prononciert souveränistischer Sicht hochgehaltene nationalstaatliche Souveränität und scheinbar unantastbare Grundsätze der Nichteinmischung geworden sind. Einer globalen Umweltverfassung dürften also weniger rechtssystematische als politische Gründe im Wege stehen.

Ein nicht unbedeutender Fortschritt ist das Escazú-Agreement von 2020, das auf der vor 20 Jahren beschlossenen Aarhus-Konvention aufbaut und bisher von elf lateinamerikanische Staaten ratifiziert wurde. Es verbessert nicht nur die multilaterale Koordination der Umweltpolitik, sondern schafft auch bessere Zugänge demokratischer Information und Beteiligung und senkt die Schwelle für Sammelklagen indigener Stämme und von Menschenrechtsaktivisten vor nationalen Gerichten (Maihold und Reisch 2021). Man erkennt dabei, welche Parallelaktionen mit Urteilen internationaler Gerichtshöfen und möglicherweise auch Lieferkettengesetzen einzelner Staaten und der Europäischen Union denkbar sind, damit sich ein regional differenziertes transnationales Umweltrecht aus beiden Richtungen von oben und unten aufbauen kann.

## Posthumane Internationale Beziehungen?

Das von dem Anfang 2021 verstorbenen Chemiker und Erdsystemforscher Paul Crutzen eingeführte Konzept des Anthropozän hat die Politikwissenschaft erreicht. Die Erkenntnisse der zunehmend interdisziplinären Erdsystemwissenschaft erlauben kein „Business as usual“ mehr (Arias-Maldonado und Trachtenberg 2019), da das vorfindliche globale Vertragssystem die Bedrohung durch Klimawandel und Artensterben nicht aufzuhalten vermochte. Startpunkt der International Relations (IR) waren die mit dem Klimawandel verbundenen Sicherheitsprobleme, die (lange ignoriert) bei grenzüberschreitenden Ressourcenkonflikte und Massenmigrationsbewegungen aufgetreten waren und weiter auftreten können. Ein Sammelband der auf „Global Security“ spezialisierten Online-Zeitschrift „E-international Relations“ hat dabei auch die Herausforderung des „Posthumanismus“ angenommen, d. h. einer nicht vornehmlich auf den Menschen fixierten Internationalen Politik unter Einbezug *nicht-menschlicher* Komponenten und Akteure. Einige AutorInnen vertreten einen radikalen Posthumanismus, um der belebten und auch der unbelebten Natur im Sinne eines „neuen Materialismus“ (Bennett 2010) zu ihrem Recht zu verhelfen, erkenntnistheoretisch die Position einer „objekt-orientierten Ontologie“. Andere plädieren für eine „reflexive Anthropozentriertheit“ (Audra Mitchell 2017, S. 3), die menschliche Akteure für die natürliche Umwelt sensibilisiert, ihnen aber die zentrale Handlungsmacht belässt. Aus dem Anthropozän werden ganz unterschiedliche Konsequenzen gezogen: Im „good anthropocene“ können menschengemachte Umweltbelastungen und -schäden noch durch menschliche Aktion und Ingeniosität, oft in großtechnischen Skalen wie beim Geoengineering, repariert werden, „tiefenökologische“ Versionen lassen in aller Demut oder Zuversicht (und mit ungewissem Ausgang) eher dem Mitspieler Natur den Vortritt. Die klassische „cartesianische“ Dualität von Natur und Kultur, Mensch und Umwelt steht damit jedenfalls in Frage. Ob und wie dies auf die Akteursebene der Internationalen Politik durchschlägt, ist die Frage; ausschlaggebend wird die Validität und Überzeugungskraft empirischer Forschung sein, die in den von konzeptioneller Arbeit geprägten Beiträgen oft erst angedeutet werden.

Appelle zu einer „Soziologisierung“ des Anthropozän-Denkens (vgl. Adloff und Neckel 2020) finden ein Pendant in Aufrufen zur „Geologisierung des Sozialen“ (Clark und Szerszynski 2020; Hanusch et al. 2021). Die beide in Lissabon und Coimbra lehrenden PolitikwissenschaftlerInnen Joana Castro Pereira und André Saramago versuchen mit ihrem Band dezidiert den Brückenschlag (oder eine „Assemblage“) zwischen der anthropozentrischen Tradition der IR und den nicht-menschlichen Komponenten des Erdsystems. Ziel ist eine „interspecies politics“, die auch in diesem Band eher noch angedeutete als schon gründlich ausgeführte Probleme ontologischer, epistemologischer und ethischer Natur aufwirft. Dem Postulat einer „quantum social theory“ nähern sich einige Fallstudien zur Verschlingung menschlicher und außermenschlicher Verursachungen internationaler Beziehungen wie der Klimakrise, dem Automobilverkehr und dem Brexit an. Ein Beispiel ist Rafi Youatts Versuch (2020, S. 73ff.), Berge und Gebirge nicht-reduktionistisch, also nicht als bloße Objekte von Politik zu betrachten; weitere Beiträge widersprechen dem „Ökomodernismus“ (Theorien und Pfaden sozial-ökologischer Modernisierung) und werten nicht-menschliche Wirkungsmächte prozesssoziologisch auf. Der Band enthält auch Fallstudien zur Umweltpolitik Indonesiens, der VR China und der USA und Betrachtungen zum Versuch der Operationalisierung von „Rechten der Natur“ durch die Europäische Union und Aktivisten von ENGO’s („Environmental Non-Governmental Organizations“) wie dem World Wildlife Fund und der Intergovernmental Science-Policy Platform on Biodiversity and Ecosystem Services (IPBES).

So verschwommen die Terminologie und so fragmentiert die alternativen Politikansätze bei skeptischer Betrachtung bleiben, so wichtig und bereichernd erscheinen diese Landgewinne der IR in der Summe Fachvertretern wie dem in Kopenhagen lehrenden Olaf Corry (S. 337ff.) Das Studium der Internationalen Beziehungen lässt sich nicht länger auf eine rein sozial- und geisteswissenschaftliche Methodik und Empirie beschränken, mit dem Anschluss an die Erdsystemwissenschaft wandern Erkenntnisse über die nicht-menschliche Natur und deren Wirkmechanismen ein, die nichts so schlagartig unter Beweis gestellt hat wie die SARS-CoV-2-Pandemie, die in der angezeigten Literatur zwar noch keine Rolle spielt, aber künftige Forschung inspirieren wird. Am stringentesten erscheinen die Ansätze des in Utrecht lehrenden Politikwissenschaftlers Frank Biermann, der als einer der ersten die Reichweite von „Global Governance“ zum Thema „Earth System Governance“ (2014) geöffnet und sich nun, im Blick auf die Dringlichkeit globalen Handelns, weiter radikalisiert hat. Mit der in Linköping tätigen Kollegin Eva Lövbrand hat er ein Kompendium grünen politischen Denkens vorgelegt, was nicht eng parteipolitisch gemeint ist, sondern als Konsequenz ein umweltpolitisches Engagement reklamiert, das von den meisten ForscherInnen unter Berufung auf die Werturteilsfreiheit abgelehnt wird (Biermann und Lövbrand 2019). Doch fordern die Hybridität, Komplexität und Nicht-Linearität der Mensch-Natur-Beziehungen Grundkonzepte wie Macht und internationale Gerechtigkeit und basale Kategorien wie Raum und Zeit heraus, ebenso fest etabliert scheinende Konzepte liberaler Demokratie.

Für die Analyse Internationaler Beziehungen und Globalen Regierens ist viel gewonnen, wenn die belebte wie nicht-belebte Natur als bisher „ausgeschlossene Andere“ wenigstens gedanklich repräsentiert und virtuelle Mitglieder einer „Zoopolis“ würden. Die demokratiepolitische Kernfrage ist, ob diese Inklusion (nur) menschliche Handlungsparameter einschränken würde oder demokratische Legitimität durch „Proxy-Repräsentation“ zu erweitern wäre. Wie radikal sollen wir die Inklusion denken: Können Mensch *mit* Tieren sprechen und sie verstehen, können sie damit auch *für* Tiere sprechen – oder können Tiere* an und für sich *sprechen und auf ihre Weise an deliberativen Aushandlungen mitwirken (Meijer 2019)? Und dürfen sie dann Rechte ausüben, ohne im klassischen Sinne des Gesellschaftsvertrags auch Pflichten zu übernehmen (die Menschen ihnen ohne jede Reziprozität allerdings ohnehin aufbürden)? Hier betritt man den spekulativen Bereich demokratischer Experimente, die, wenn man so will: Bewährungsprobe planetaren Denkens, die von avantgardistischen Ansätzen wie Bruno Latours „Parlament der Dinge“ (2001) und aktuell von Eva Meijers „interspecies democracy“ (2019) angedacht, aber noch nicht operativ ausgefeilt worden ist. Die Internationale Politik hält sich zugute, im Rahmen der UN-Nachhaltigkeitsziele und des Pariser Klimaabkommens, die durch Regierungsverhandlungen unter Einschluss von NRO, indirekt auch Protestbewegungen zustande gekommen sind, einen guten Schritt voran getan zu haben. Was sollte da eine virtuelle Einbeziehung nicht-menschlicher Akteure bewirken? Die Antwort könnte sein, dass ihre Nicht-Berücksichtigung die bis dato schwache und langsame Implementierung solcher Absichtsbekundungen erklärt – oder auch, dass die mit den internationalen Abkommen verbundenen Hoffnungen ohnehin anmaßend waren und Illusionen nähren.

Der am Stockholm Resilience Centre tätige Victor Galaz betont dazu den wichtigen Aspekt der Zeit für die Herstellung internationaler Gerechtigkeit (S. 109–127). Das Beispiel der „transitional justice“ zeigt, dass Recht und Politik zumeist zurückblicken und retroaktiv vergangenes Unrecht thematisieren und wiedergutzumachen bestrebt sind. Dramatische Folgen des Klimawandels wie der Anstieg des Meeresspiegels erzwingen den Perspektivenwechsel in die Zukunft und die Befassung mit dem ungewissen Schicksal künftiger Generationen; deren Bedürfnisse und Orientierungen können Zeitgenossen so wenig bestimmen wie die der nicht-menschlichen Natur, aber man muss deren Rechte auf ein (heutigen Verantwortlichen zumindest) ebenbürtiges Leben ebenfalls respektieren und repräsentieren. Galaz arbeitet präzise heraus, wie Verhältnisse der „deep time“, der geosozialen Ur- und Frühgeschichte des Anthropozän, Zukünfte prädeterminiert, während in der Gegenwart Mechanismen der Telekommunikation, Finanzwirtschaft und Kriegsführung in „ultra-speed“ ablaufen – womit genau jene Zeitsouveränität gleich dreifach verloren geht, die im Blick auf die Kipppunkte des Erdsystems so dringend erforderlich wäre. Ayşem Mert (Stockholm/Amsterdam) (2019, S. 128–149) denkt über eine „Hochskalierung“ demokratischer Aushandlungs-prozesse auf die globale Ebene nach, die ähnlich revolutionär sein müsste wie beim Ausbau der griechischen Polis-Demokratie in die repräsentativen Demokratien seit dem 18. Jahrhunderts.

Anthony Burke und Stephanie Fishel, die „Ding-Systeme im Anthropozän“ behandeln (S. 87–108), haben auf dem Weg dahin ökologisch inklusivere Governance-Institutionen mit einer Quasi-Repräsentation nicht-menschlicher Wesen vorgeschlagen: „15 regionale Ökosystem-versammlungen zur Abdeckung der wichtigsten Biome der Erde und einen Erd-Systemrat zur Koordinierung integrierter Maßnahmen, zu denen auch die Vertretung von Staaten, indigenen Gemeinschaften und Proxies von Nicht-Menschen gehört. Solche Institutionen erfordern ein tiefes Engagement für die Komplexität und Vitalität der Biosphäre, Reflexivität und Demut für die Reparatur des Erdsystems und ein ständiges Bewusstsein für die aporetische Qualität der politischen Repräsentation als solche, um neue Formen der Politik und des Regierens ‚zwischen den Arten‘ für und mit der Biosphäre als Ganzer.“ (Burke et al. 2016, S. 33).

So verstanden impliziert ein planetarer Blick keineswegs das Ende „internationaler Politik“, aber deren Radikalisierung. Zu vermeiden sind dabei Generalisierungen des „Humanen“ genau wie des „Nicht-Humanen“. „Menschheit an sich“ ist ebenso wenig eine Akteurskategorie wie die „Natur als Ganze“, und trotz ihrer wechselseitigen Bedingtheit und Interaktionen soll eine (nicht-cartesianische) Dualität bestehen bleiben. Denn in summarischen Aussagen zur „globalen Erwärmung“ fallen (ungleiche) Sozialstrukturen und (variierende) politische Akteure oft unter den Tisch. Praktisch könnten drei Global Governance-Protagonisten am ehesten eine planetare Politik gestalten: die Europäische Union als bereits weit entwickelter supranationaler Akteur, der eine direkte demokratische Legitimation durch das Europäische Parlament und eine indirekte aus den nationalen Abgeordnetenhäusern vorweisen kann, spezieller dann Environmental Non Governmental Organizations (ENGO) und Internationale Stewardship-Vereinigungen, wie Sea Shepherd Conservation Society (SSCS) und Intergovernmental Science-Policy Platform on Biodiversity and Ecosystem Services (IPBES). Letztere ist eine UN-Organisation der wissenschaftlichen Politikberatung in 136 Mitgliedsstaaten zu Fragen der Erhaltung und nachhaltigen Nutzung der biologischen Vielfalt und von Ökosystemdienstleistungen.

Die hier in aller Kürze vorgestellten Publikationen belegen ein erneuertes Interesse am politischen Kosmopolitismus. Ein wichtiges Such- und Experimentierfeld ist die Ausarbeitung transnationaler Verfassungsgrundsätze, vor allem im Politikfeld „Umwelt“, das schon von seiner Dringlichkeit her die zugespitzte Opposition von Kosmopolitismus und Kommunitarismus anachronistisch erscheinen lässt. Dazu hat die Diffusion der Anthropozän-These beigetragen, deren Konsequenz die Einbeziehung der belebten wie unbelebten Natur als virtuellem Mitakteur internationaler Beziehungen ist. In den Horizont des Kosmopolitismus sind damit materiell, räumlich und zeitlich „ganz Andere“ geraten, was eine konzeptionelle Revision kosmopolitischer Ideen in Richtung auf eine übergreifend planetare „Kosmo-Politik“ nach sich zieht. Dazu gehört eine Neuorientierung des wissenschaftlichen Selbstverständnisses, das Silke Beck (Helmholtz-Zentrum für Umweltforschung Leipzig) und andere in der Forschungsplattform „Future Earth: Research for Global Sustainability“ unter Einbeziehung von Laien und politischen Akteuren erproben (S. 191–211). Dem Mainstream (nicht nur) der Politikwissenschaft werden diese gleich mehrfachen Grenzüberschreitungen – von der Staatenordnung der Weltgesellschaft in den Kosmos der nicht-menschlichen Natur, von der Sozial- in die Erdsystemwissenschaft und von der „reinen“ Wissenschaft in die Domäne der „Citizen Science“ – womöglich nicht behagen und schwer einleuchten. Das gilt umso mehr für den Übergang vom Anthropozentrismus zum „Ökozentrismus“ und zur Einnahme einer planetaren Perspektive (jetzt Chakrabarty 2021; Chandler et al. 2021). Dass die Menschheit nach den Erkenntnissen der Erdsystemwissenschaft an einem regelrechten Abgrund stehe, erfordert eine neue, nunmehr planetare Kritische Theorie, die den Kosmopolitismus in eine Kosmo-Politik neuen Typs fortschreibt.

## Footnotes

1 „Ich möchte *Weltbürger* sein, überall zu Hause und, was noch entscheidender ist, überall unterwegs.“ Brief an Marcus Laurinus, 1. Februar 1523, in: P.S. Allen (Hrsg.) Opus Episolarum Bd. V, Oxford 1924, S. 539.

2 http://hrlr.law.columbia.edu/hrlr-online/prosecuting-international-environmental-crime-committed-against-indigenous-peoples-in-brazil/.
